# Enterococci Isolated from Trout in the Bukovec Water Reservoir and Čierny Váh River in Slovakia and Their Safety Aspect

**DOI:** 10.1155/2019/8051438

**Published:** 2019-11-29

**Authors:** Andrea Lauková, Ivana Kubašová, Eva Bino, Anna Kandričáková, Viola Strompfová, Rudolf Žitňan, Monika Pogány Simonová

**Affiliations:** ^1^Institute of Animal Physiology, Centre of Biosciences of the Slovak Academy of Sciences, Laboratory of Animal Microbiology, Šoltésovej 4-6, 040 01 Košice, Slovakia; ^2^National Agricultural and Food Centre, Research Institute for Animal Production, Hlohovecká 2, 951 41 Nitra-Lužianky, Košice, Slovakia

## Abstract

The aim of this study was to investigate enterococci as lactic acid bacteria and as part of Firmicutes phylum. We focused on the virulence factor, biofilm formation, and antibiotic resistance and also on lactic acid production and enterocin gene detection. Intestinal samples were taken from 50 healthy trout (3 *Salmo trutta* and 47 *Salmo gairdneri*) collected in April 2007, 2010, and 2015 from different locations at the Bukovec water reservoir and the Čierny Váh River in Slovakia. Twenty pure colonies were identified using the matrix-assisted laser desorption/ionization time-of-flight mass spectrometry identification system based on protein fingerprints, and then seven identified strains were also phenotyped. Based on the identification methods used, the identified enterococci (7) belong taxonomically to four different enterococcal species: *Enterococcus durans*, *E. faecium*, *E. mundtii*, and *E. thailandicus.* They were hemolysis, DNase, and gelatinase negative with acceptable enzymatic activity. They did not form biofilm and were mostly susceptible to antibiotics. All strains produced lactic acid amounting to 1.78 ± 0.33 mmol/l on average and possessed the gene for enterocin A production. This is the first study reporting more detailed properties of enterococci from trout in Slovakian wild water sources, and it produces new possibilities for studying microbiota in trout.

## 1. Introduction

Aquatic sources and/or aquaculture are increasingly used to produce aquatic food all over the world. Fish are mostly reared in two fish farming facilities with a capacity of 140.503 m^3^ and in 485 fish pools covering an area of about 2000 Ha [1, 2]. In Slovakia, trout is the most popular food fish, and aquaculture can be classified into two groups: fish farm and lowland wild fish species [2]. In general, the microbiota in trout from fish farms is more studied. There is for instance new information regarding the lactic acid bacteria (LAB) in trout from a commercial fish farm [2, 3], but limited data are available regarding trout from wild sources. Different LAB have adapted to grow under widely different environmental conditions, and they are widespread in nature. Fish are exposed to a wide range of microorganisms present in the environment. Ringø and Gatesoupe [4] demonstrated that the genera *Streptococcus, Leuconostoc*, *Lactobacillus*, *Enterococcus*, and *Carnobacterium* belong to the normal microbiota of the gastrointestinal tract (GIT) in healthy fish. Didinen et al. [3] identified the species *Lactobacillus sakei* and *Lactococcus lactis* subsp. *cremoris* or subsp. *lactis* in rainbow trout from farms in Turkey using 16S rRNA gene sequence analysis. It has also been reported that some LAB isolated from the GIT of fish can act as probiotics [3, 5, 6]. These candidates are able to colonise the gut and act as antagonists against Gram-negative fish pathogens [3, 5]. Some of these bacteria can also produce bacteriocins, i.e., antimicrobial proteinaceous substances. Araújo et al. [7] evaluated enterococci from rainbow trout, their feed, and rearing environment with inhibition potential against fish pathogens. Enterococci can produce enterocins. Known enterocins are produced mostly by strains representing the species *Enterococcus faecium* [8]. However, also other enterococcal species were detected to produce enterocins [9]. We were focused on enterococci in trout from wild sources. The aim of this study was to check for enterococcal strain benefits in fish such as lactic acid production or *enterocin* genes; we tested for properties of enterococci to contribute to basic microbiology but also to select a potential candidate for inhibiting undesirable bacterial agents in trout. Beneficial strains for use in aquaculture should be regarded as safe, not only for the aquatic hosts but also for their surrounding environment and for humans (consumers) [2, 5]. Finally, our intention was preliminary studying enterococci on virulence factor parameter, biofilm formation, and antibiotic resistance regarding the safety aspect.

## 2. Materials and Methods

### 2.1. Sample Collection

Intestines were taken from 50 healthy trout (3 *Salmo trutta* and 47 *Salmo gairdneri*) collected in April 2007, 2010, and 2015 from different locations at the Bukovec water reservoir near Košice in eastern Slovakia and the Čierny Váh River in central Slovakia. They were sampled at the point of collection of the trout, stored at 4°C for approximately 4 h, and transported to the laboratory. After delivery, the samples were treated using the standard microbial dilution method (International Organization for Standardization, ISO); they were stirred (1 : 9) in Ringer solution (pH 7.0, Merck, Germany); appropriate dilutions were plated onto cultivation medium M-Enterococcus agar (Difco, Detroit USA) to count colonies of enterococci. Plates were cultivated at 37°C for 48 h. Grown colonies (those from the highest dilution) on M-Enterococcus agar were randomly picked up and checked for purity by plating on Brain Heart Agar enriched with blood (BHA, Difco, USA) to check their growth—Gram stain morphology; then they were plated for further tests. The Microbank system (Pro-Lab Diagnostic, Richmond, Canada) was used to store identified strains.

### 2.2. Strain Identification

Twenty pure colonies were identified using the matrix-assisted laser desorption/ionization time-of-flight mass spectrometry (MALDI-TOF MS) identification system based on protein fingerprints (Bruker Daltonics) [10] and performed using a Microflex MALDI-TOF MS mass spectrometer as described in the previous study by Lauková et al. [11]. A pure single colony from BHA enriched with blood was mixed with matrix (*α*-cyano-4-hydroxycinnamic acid and trifluoroacetic acid), and the suspension was spotted onto a MALDI plate and ionized with a nitrogen laser (wavelength of 337 nm and frequency of 20 Hz). Results were evaluated using the MALDI BIOTYPER 3.0 (Bruker Daltonics USA) identification database. Taxonomic classification was evaluated on the basis of highly probable species identification (value score 2.300–3.000) and/or secure genus identification/probable species identification (2.000–2.299). Positive controls were those provided in the identification system. Identical colonies evaluated on the basis of MALDI-TOF MS score values were excluded. Finally, seven strains were submitted for further testing.

The strains were also phenotyped using commercial BBL Crystal Gram-positive ID System kit (Becton and Dickinson, Cockeysville, USA); the control strains were those included and recommended in the kit. This kit includes hydrolysis of urea, esculin, and arginine, hydrolysis of enzymes, and utilization of carbohydrates (trehalose, lactose, sucrose, mannitol, fructose, arabinose, etc.). In addition, fermentation of melibiose and galactose was tested.

### 2.3. Enzymatic Activity (API ZYM) and Lactic Acid Production

To evaluate the functionality/safety of strains, enzymatic activity was tested. The API ZYM tests (BioMériux, France) containing 19 different substrates were used. Suspensions of strains with a turbidity of one McFarland were prepared in 200 *μ*l of sterile distilled water; 65 *μ*l of suspensions was dispensed into each well with substrate. After inoculation, strains were incubated for 4 h at 37°C in incubation boxes provided by the test supplier. Then a drop of ZYM A and ZYM B reagent was added to each well, and reactions were read after exposure to light for a few seconds. The color intensity of the reaction was estimated in the range from 0 to 5 corresponding to the activity from 0 to 40 nmol.

Enterococci belong to lactic acid bacteria of the Firmicutes phylum; for this reason, production of lactic acid (LA) was analysed using the validated spectrophotometric method and measured at 565 nm (Specol 11, Carl Zeiss, Jena, Germany) as previously described by Lauková et al. [12]. This method is based on the conversion of lactic acid to acetaldehyde by heating with sulfuric acid. Acetaldehyde reacts with 4-hydroxybiphenyl, forming a color complex. The LA amount is expressed in millimole per liter (mmol/l).

### 2.4. Enterocin Gene Detection Using PCR

Some enterococci are known to produce antimicrobial proteinaceous substances possessing genes for their production. In this study, *enterocin* genes for six enterocins were checked: ent A, ent P, ent B, ent L50A, L50B, and ent 31. They were selected based on our previous studies [13]. Primer sequences for PCR amplification of *ents* genes were used according to Aymerich et al. [14], for ent A according to Cintas et al. [15, 16], and for ent P, L50A and L50B, and ent 31 according to De Vuyst et al. [17]. PCRs were carried out using a C1000™ thermal cycler (Bio-Rad Laboratories, Hercules, USA). PCR product was visualized by means of electrophoresis in 2% agarose gels (Sigma-Aldrich) buffered with 1x Tris-acetate-EDTA buffer (Merck) and 1 *μ*g ethidium bromide. Positive control strains were *E. faecium* EK13/CCM7419 [18] for ent A and P and *E. faecium* L50 [15–17] for ent L50B, L50A, and ent 31. A template was added to the reagent mixture (25 *μ*l) containing 1x reagent buffer, 0.2 mmol/l dNTPs (deoxynucleotide triphosphate) (Invitrogen), 1 *μ*mol/l of each primer, 1 U Taq polymerase, template, and water. DNA (template) was extracted by applying the rapid alkaline lysis method [19]. The cycle for ent A and ent 31 was as follows: denaturation at 95°C for 5 minutes, followed by 30 cycles at 95°C for 30 sec, then 58°C for 30 sec, 72°C for 30 sec, and finally 72°C for 5 min. The cycle for ent P, L50A, B, and L50B differed with the temperature used 56°C instead of 58°C.

### 2.5. Determination of Hemolytic, Gelatinase, and Nuclease Activity

To exclude virulence of strains, some parameters such as hemolysis, gelatinase, and nuclease activity were tested. Hemolysis activity was tested by streaking the cultures on De Man–Sharp–Rogosa (MRS) agar (Difco, Detroit USA) supplemented with 5% defibrinated sheep blood. Plates were incubated at 37°C for 24–48 h under semianaerobic conditions. The presence and absence of clearing zones around the colonies were interpreted as *α*, *β*-hemolysis and negative *γ*-hemolysis, respectively [20].

Gelatinase activity was tested with a 3% gelatin medium (Todd Hewitt agar, Becton and Dickinson, Cockeysville, Maryland, USA) according to Semedo-Lemsaddek et al. [20]. The loss of turbidity halos around colonies of tested strains was checked at 4°C.

Each identified strain was inoculated on the surface of deoxyribonuclease agar (DNase agar, Oxoid, USA). The production of DNase was evaluated after 24 h incubation at 37°C. Colonies producing DNase hydrolysed the deoxyribonucleic acid contained in the medium. After agar flooding and acidifying with 1N HCl (hydrochloric acid), the DNA precipitated, and the medium became turbid with clearing zone formation around DNase-positive colonies.

### 2.6. Biofilm Formation

The ability of enterococci to form biofilm is a parameter belonging to the group of virulence factors. Biofilm formation was assessed with a quantitative plate assay according to Chaieb et al. [21]. In brief, one colony of the tested strain grown overnight at 37°C on Trypticase soy agar (Difco, Michigan, USA) was transferred into 5 ml of Ringer solution (pH 7.0, 0.75% w/v) to obtain concentration cells in suspension corresponding to 1 × 10^8^ CFU/ml. A volume of 100 *μ*l from that culture was then transferred into 10 ml of Trypticase soy broth (TSY). That standardized culture (200 *μ*l) was inoculated in a well on a polystyrene microtiter plate (Greiner ELISA 12 Well Strips, 350 *μ*l, flat bottom, Frickenhausen GmbH, Germany) and incubated for 24 h at 37°C. The biofilm which was formed in the microtiter plate well was washed twice with 200* μ*l of deionized water and dried at 25°C for 30 min in an inverted position. The remaining attached bacteria were stained for 30 min at 25°C with 200 *μ*l of 0.1% (m/v) crystal violet in deionized water. The dye solution was aspirated away, and the wells were washed twice with 200 *μ*l of deionized water. After water removal and drying for 30 min at 25°C, the dye bound to the adherent biofilm was extracted with 200 *μ*l of 95% ethanol and stirred. A 150 *μ*l aliquot was transferred from each well and placed on a new microtiter plate for optical density-absorbance (OD-A) determination at 570 nm using a Synergy TM4 Multimode Microplate reader (Biotek, USA). Each strain and condition was tested in two independent tests with 12 replicates. Moreover, a sterile culture medium was included in each analysis as a negative control. *Streptococcus equi* subsp. *zooepidemicus* CCM 7316 was used as a positive control in each method (kindly provided by Dr. Eva Styková, University of Veterinary Medicine and Pharmacy, Košice, Slovakia, [22]). Biofilm formation was then classified as highly positive (A_570_ ≥ 1), low-grade positive (0.1 ≤ A_570_ < 1), and negative (A_570_ < 0.1).

### 2.7. Antibiotic Susceptibility or Resistance Testing

Knowing the reaction of strains to antibiotics is one of the diagnostic parameters as well as a factor regarding the safety of strains because of resistance elements. Antibiotic susceptibility/resistance testing in identified enterococci (100 * μ*l of an 18 h culture of each strain) was tested using the qualitative agar disc diffusion method on Columbia agar (Becton and Dickinson) enriched with 10% of defibrinated sheep blood and on Mueller-Hinton agar (Difco) according to Clinical and Laboratory Standards Institute method-CLSI 2016 [23]. Thirteen antibiotic discs (Oxoid, Basingstoke, United Kingdom) were applied: clindamycin (DA-2* μ*g), novobiocin (Nb-5* μ*g), ampicillin (AMP-10* μ*g), penicillin (P-10IU), erythromycin (E-15* μ*g), azithromycin (AZM-15* μ*g), streptomycin (STR-25* μ*g), chloramphenicol (C-30 * μ*g), rifampicin (RA-30 * μ*g), tetracycline (TC-30* μ*g), vancomycin (VAN-30* μ*g), kanamycin (KAN-30* μ*g), and gentamicin (GN-120* μ*g). After incubation at 35 (37)°C overnight, the strains were evaluated as resistant or susceptible according to the manufacturers' instruction; the inhibition zone was expressed in millimeter. Antimicrobial free agar plates were included as a control for obligatory strain growth. The use of the antimicrobial agents was decided according to the manufacturers' recommendation (Oxoid) and the most relevant antibiotics for enterococci from clinical view. *Enterococcus faecium* CCM 4231 was used as a positive control [24].

## 3. Results and Discussion

The total enterococcal count from the GIT of 50 trout was 1.40 ± 0.71 CFU/g (log_10_) on average. Twenty colonies grown on agar (one sample from each) were picked up, and among those 20 colonies, seven strains were finally identified as belonging taxonomically to four different enterococcal species, namely, *Enterococcus durans*, *E. faecium*, *E. mundtii*, and *E. thailandicus*. The rest of the strains were not identified; they represented identical colonies, respectively (they were excluded from further testing). Two strains (both *E. faecium*) were evaluated as reaching a score which corresponded with highly probable species identification (2.300–3.000, Table 1). Five strains were evaluated with scores related to secure genus identification/probable species identification (2.000–2.299, Table 1). Phenotypic properties were compared with those for reference strains in Bergey's Manual of Determinative Bacteriology [25] and according to Tanasupawat and Sukontasing [26], respectively. They showed for instance positive reaction for galactose in *E*. *thailandicus*, *E. faecium*, *E. mundtii*, and *E. durans*. Similarly, positive reaction for xylose was found in *E. mundtii* and negative in *E. faecium* and *E. durans*. Fructose, lactose, and trehalose tests were evaluated as positive. Melibiose was fermented in *E. thailandicus* and *E. mundtii*; on the other hand, melibiose was not fermented in *E. durans*, and a dubious reaction was evaluated in *E*. *faecium*. Fermentation testing with sorbitol was mostly negative or dubious. Mannitol testing was positive in *E. durans*, *E. thailandicus*, and *E. mundtii* and dubious in *E. faecium*. Maltose was fermented (positive).

Regarding enzymatic activity, our strains showed low or 0 values in relation to trypsin and *α*-chymotrypsin, but also in relation to *β*-glucuronidase and other enzymes. All strains reached 10 mmol in the case of naphthol-AS-BI-phosphohydrolase (Table 2). Higher values appeared in the case of *β*-glucosidase, and the highest value measured, 20 mmol, was in strains EFR39/2 and ETR29/1.

LA production was high (Table 1), 1.53 ± 0.66 mmol/l on average. It was also well balanced, not depending on the species.

Among the six *ent* genes tested, only one-*ent* A gene was confirmed in all enterococcal species (Table 3). Enterococci were free of the other *enterocin* genes tested.

The enterococci detected were free of virulence factor phenotype such as hemolysis; they were DNase negative and gelatinase negative as well (Table 1). Moreover, four strains tested for biofilm formation did not form biofilm (Table 3). The values of absorbance (A_570_) measured were less than 0.1.

The enterococci were mostly susceptible to the tested antibiotics (ATB) except kanamycin and gentamicin, which are chromosomally encoded in enterococci. This means that the strains EMR39/1, EFR38/2 EF35R/1, and ETR29/1 were monoresistant (Table 4), while *E. durans* EDR38/1was resistant to four ATBs and *E. durans* EDR36/1 was resistant to three ATBs, as well as strain EFR39/2. Strains were also resistant to streptomycin, and two were resistant to TC, Nb, and AZM, one in each strain. All strains were susceptible to VAN, P, AMP, E, C, and RA.

Enterococci are lactic acid bacteria comprising both pathogenic and commensal microorganisms ubiquitous in the environment, even as gut symbionts [27]. Although in fish detected enterococci did not participate in high amount, they are a part of the lactic acid bacteria (LAB); LAB such as lactococci or lactobacilli and also *E. faecium* were previously detected in fish [7, 28]. Detection of four different species among the seven identified enterococci from trout indicated their high species variability. It is interesting that the species detected belong to the same group (*E. faecium*) based on 16S rRNA gene similarity [29]. The MALDI-TOF identification system was successfully applied to the identified bacteria especially for research [30]. In this study, enterococci were detected with high identification score. *E. faecium* or *E. faecalis* are usually the most frequently detected enterococcal species either in the GIT or in the faeces of animals [31, 32]. *E. mundtii*, *E. durans*, or *E. thailandicus* are rarely isolated from animals' GIT. However, Lauková et al. [33, 34] isolated *E. mundtii* from pheasants and ostriches and *E. thailandicus* from the faeces of beavers. Moreover, as mentioned above, all detected species belong to the same group (*E. faecium*) based on 16S rRNA gene similarity [29].

Regarding enzymatic activity, the enterococci detected showed zero value in the case of enzymes trypsin and *α*-chymotrypsin which are usually associated with intestinal disease for example. They did not show protease activity. Moreover, the values of *β*-glucuronidase were 0 or 5 nmol (in strain EFR39/1), which is beneficial. For example, in humans, *β*-glucuronidase is an enzyme which can serve as a cancer marker [35]. Following the benefit of enzymatic testing, the values of *β*-glucosidase were not so high either. However, they were sufficiently high to have beneficial effect, for instance in lactose fermentation. Compared with the enterococci from aquaculture presented by Araújo et al. [7], the enzymatic activity values in this study were lower.

Lactic acid is a metabolic product of enterococci. Similar amounts of LA as in enterococci tested (1.78 ± 0.33 mmol/l, on average) were measured in enterococci isolated from the faeces of pheasants [33]. LA could also contribute to antimicrobial activity in the tested strains.

Among enterocins, only the gene for *ent* A was confirmed in all enterococcal strains. The gene most frequently detected in enterococci is *ent* P followed by e*nt* A [13]. The reason why enterococci have developed ability to produce these antimicrobial peptides (bacteriocins-enterocins) is unknown. It is possible that bacteriocin production is a beneficial probiotic trait in some environments [36]. One surprising point in this study was the detection of *ent* A gene in various species of enterococci, although the one described in the first instance was produced by *E. faecium* [8].

The strains were DNase negative. Similarly, in rabbit faeces, *E. faecalis* and *E. faecium* strains were detected, which were also DNase negative [37]. If enterococci are found to be hemolytic, then *α*-hemolysis is more typical. However, enterococci in this study did not form hemolysis. In addition, they were also gelatinase negative (but we did not test for the presence of any gene). It could be necessary to test for the occurrence of the *gelE* gene as well because in some cases the gelatinase-positive phenotype does not mean the presence of that gene, as reported for example in enterococci of Pannon White breed rabbits faeces by Lauková et al. [37]. On the other hand, the gelatinase-negative phenotype in a strain can harbour the gene. However, Araújo et al. [7] for instance confirmed the *gelE* gene presence in 46.9% of enterococci (64 strains) isolated from rainbow trout, their feed, and the rearing environment. Biofilm formation is assumed to be a factor of pathogenicity because it can serve as a protective barrier against host defences and the action of antimicrobials; thus, it could be a possible source for persistent infection [38]. However, the enterococci we tested did not form biofilm. On the other hand, it is known that *E. hirae* isolated from various animals produced biofilm [38].

As previously mentioned, enterococci are chromosomally resistant to KAN, which was also confirmed in this study. They were mostly susceptible to ATBs. All were susceptible to VAN, as also previously reported by Migaw et al. [39] in enterococci isolated from Mediterranean fish viscera.

As in every part of present-day life, there is interest in probiotic strains or their products in aquaculture as well. It has been found that beneficial (probiotic) organisms can also improve water quality in aquaculture ponds because probiotic bacteria are able to participate in the metabolizing of organic nutrients in the water [40]. In the next, these enterococci will be tested for their bacteriocin (antimicrobial) activity because they have *ent* A gene. They did not have any of the virulence factors we tested for (using phenotype), which is promising in terms of their possible application. In future, we plan to test the bacteriocin activity of those strains against fish pathogens. However, to confirm their safety, genes for virulence factors will be tested and resistance genes as well. In each case, whether probiotic or bacteriocin activity, this study is original as it provides basic knowledge for subsequent, more detailed studies of individual strains and their antimicrobial substances. These are its contributions to aquatic and aquaculture microbiology.

## 4. Conclusion

Based on the identification methods used, enterococci belong taxonomically to four different enterococcal species, namely, *Enterococcus durans*, *E. faecium*, *E. mundtii*, and *E. thailandicus.* They were hemolysis, DNase, and gelatinase negative; they did not form biofilm and were mostly susceptible to antibiotics. All strains possessed the gene for enterocin A production, and they produced lactic acid amounting to 1.78 ± 0.33 mmol/l on average with acceptable enzymatic activity. This is the first study reporting in more detail the properties of enterococci from trout, from “wild sources” in Slovakia, and it produces new possibilities for studying microbiota in trout.

## Figures and Tables

**Table 1:** Species identification, hemolysis, nuclease activity, gelatinase, and lactic acid production in enterococci from trout.

Strain	Species	MALDI score	Hem	DNase	Gel	LA
R36/1	*E. durans*	2.201	ng	ng	ng	1.43 (0.69)
R38/1	*E. durans*	2.148	ng	ng	ng	1.44 (0.69)
R35/1	*E. faecium*	2.409	ng	ng	ng	1.50 (0.66)
R38/2	*E. faecium*	2.400	ng	ng	ng	1.63 (0.62)
R39/2	*E. faecium*	2.182	ng	ng	ng	1.53 (0.65)
R39/1	*E. mundtii*	2.201	ng	ng	ng	1.70 (0.59)
R29/1	*E. thailandicus*	2.273	ng	ng	ng	1.45 (0.69)

MALDI-TOF score: highly probable species identification (value score 2.300–3.000) and/or secure genus identification/probable species identification (2.000–2.299). Hem: hemolysis negative; ng: negative; DNase: deoxyribonuclease, Gel: gelatinase; LA: lactic acid expressed in mmol/l ± SD.

**Table 2:** Enzymatic activity in identified enterococcal species (in nmol).

		R36/1	R38/1	R35/1	R38/2	R39/2	R39/1	R29/1
1		ng	ng	ng	ng	ng	ng	ng
2		5	5	5	5	5	5	0
3		10	10	10	5	10	5	10
4		10	10	10	5	10	5	5
5		0	0	0	0	0	5	0
6		5	10	10	5	20	5	0
7		5	0	0	0	0	0	0
8		5	5	0	0	5	5	0
9		0	0	0	0	0	0	0
10		0	0	0	0	0	0	0
11		0	0	5	5	0	0	0
12		10	10	10	10	10	10	10
13		5	5	5	0	5	0	0
14		5	0	0	0	0	5	5
15		0	0	0	0	0	5	0
16		5	0	5	0	0	0	5
17		5	5	10	10	20	10	20
18		0	5	5	5	5	0	5
19		5	0	0	0	0	0	5
20		0	0	0	0	0	5	0

(1) Control, (2) alkaline phosphatase, (3) esterase, (4) esterase-lipase, (5) lipase, (6) leucine, (7) valine, (8) cysteine, (9) trypsin, *α*-chymotrypsin, (11) acid phosphatase, (12) naphthol-AS-BI-phosphohydrolase, (13) *α*-galactosidase, (14) *β*-galactosidase, (15) *β*-glucuronidase, (16) *α*-glucosidase, (17) *β*-glucosidase, (18) N-acetyl-*β*-glucosaminidase, (19) *α*-mannosidase, and (20) *α*-fucosidase.

**Table 3:** Detection of *enterocin* genes and biofilm formation in enterococci from trout.

Strain	*Ent* gene	Biofilm
EDR36/1	+	0.051 ± 0.03
ED38/1	+	Nt
EFR35/1	+	0.001 ± 0.00
EFR38/2	+	0.020 ± 0.03
EFR39/2	+	0.007 ± 0.02
EMR39/1	+	Nt
ETR29/1	+	Nt

+ refers to presence of *enterocin* A gene. Genes for ent P, B, L50A, L50B, and Ent 31 were not present in tested strains. Nt: not tested.

**Table 4:** Testing for antibiotic resistance among enterococci from trout.

GM	DA	Nb	AMP	P	E	AZM	STR	KAN	C	VAN	RA	TC
7/7	7/1	7/1	7/0	7/0	7/0	7/2	7/3	7/7	7/0	7/0	7/0	7/2

*x*/*x*: number of tested strains/number of resistant strains; 0: tested strains were susceptible to antibiotic used; 7/1: seven tested strains and 1 strain was resistant to tested antibiotic; 7/2: out of 7 tested strains, 2 strains were resistant to tested antibiotic; 7/3: out of 7 tested strains, 3 were resistant to tested antibiotic; DA (2 *μ*g): clindamycin; Nb (5 *μ*g): novobiocin; AMP (10 *μ*g): ampicillin; P: penicillin (10 International unit, IA); E (15 *μ*g): erythromycin; AZM (15 *μ*g): azithromycin; STR (25 *μ*g): streptomycin; KAN (30 *μ*g): kanamycin; C (30 *μ*g): chloramphenicol; VAN (30 *μ*g): vancomycin; RA (30 *μ*g): rifampicin; TC (30 *μ*g): tetracycline; GM (120 *μ*g): gentamicin.

## Data Availability

The data used to support the findings of this study are available from the corresponding author upon request.
